# TMA Navigator: network inference, patient stratification and survival analysis with tissue microarray data

**DOI:** 10.1093/nar/gkt529

**Published:** 2013-06-11

**Authors:** Alexander L. R. Lubbock, Elad Katz, David J. Harrison, Ian M. Overton

**Affiliations:** ^1^MRC Human Genetics Unit, MRC Institute of Genetics and Molecular Medicine, University of Edinburgh, Western General Hospital, Crewe Road, Edinburgh EH4 2XU, UK, ^2^Division of Pathology, University of Edinburgh, Western General Hospital, Crewe Road, Edinburgh EH4 2XU, UK and ^3^School of Medicine, University of St Andrews, North Haugh, St Andrews KY16 9TF, UK

## Abstract

Tissue microarrays (TMAs) allow multiplexed analysis of tissue samples and are frequently used to estimate biomarker protein expression in tumour biopsies. TMA Navigator (www.tmanavigator.org) is an open access web application for analysis of TMA data and related information, accommodating categorical, semi-continuous and continuous expression scores. Non-biological variation, or batch effects, can hinder data analysis and may be mitigated using the ComBat algorithm, which is incorporated with enhancements for automated application to TMA data. Unsupervised grouping of samples (patients) is provided according to Gaussian mixture modelling of marker scores, with cardinality selected by Bayesian information criterion regularization. Kaplan–Meier survival analysis is available, including comparison of groups identified by mixture modelling using the Mantel-Cox log-rank test. TMA Navigator also supports network inference approaches useful for TMA datasets, which often constitute comparatively few markers. Tissue and cell-type specific networks derived from TMA expression data offer insights into the molecular logic underlying pathophenotypes, towards more effective and personalized medicine. Output is interactive, and results may be exported for use with external programs. Private anonymous access is available, and user accounts may be generated for easier data management.

## INTRODUCTION

Oncogenic selection manifests through dysregulated pathways (1). Protein abundance and post-translational modifications (PTMs) are key determinants of network/pathway activity; therefore, functional proteomics is particularly important for understanding signalling networks underlying cancer progression, including evolution of drug resistance and metastasis (2). Tissue microarrays (TMAs) enable study of protein (and RNA) expression in *ex vivo* material, typically formalin-fixed paraffin-embedded tissue obtained at operation (3). Multiplexed immunohistochemical analysis across arrays of tissue cores efficiently derives protein expression measurements for many specimens (4). TMAs also provide greater consistency than whole section approaches due to simultaneous processing of multiple samples in identical conditions, among other features (5). Clinical subtyping frequently uses TMAs, for example to determine estrogen receptor-α (ER-α) and HER2/neu status in breast cancer (5–7). Although alternative techniques afford greater throughput for estimating protein expression, notably reverse phase protein arrays (8) and mass spectrometry (9), TMAs have particular advantages. These include identification of marker subcellular localization and discrimination of tumour compartments (e.g. stroma) using little material and without requirement for laser capture microdissection or cell fractionation (10,11). Furthermore, TMAs provide potential to identify single cell expression distributions (12). TMA Navigator provides an integrated platform for TMA data, designed to handle both categorical, semi-continuous and continuous scoring, e.g. (13–16). User-friendly interactive access is provided for data processing, investigation of marker networks and risk stratification. An option is available for reduction of batch effects, which are common, for example where data are split across multiple TMA blocks (17,18). Techniques for data exploration include kernel density estimation and Gaussian mixture modelling with Bayesian information criterion regularization for unbiased cluster identification. Analysis of survival is included (19), incorporating stratification based on mixture model results. Evidence is mounting that most phenotypes are governed by complex networks (20,21). TMA Navigator provides network inference approaches applicable to TMA datasets, which typically have relatively few markers. While several resources for TMA image data processing and management exist (22–25), few user-friendly tools provide tailored workflows for data analysis and integration with clinical variables. Stanford TMA software (26) and X-tile (27) are notable, but provide comparatively restricted functionality. Study of marker relationships in clinical samples contributes to the development and testing of hypotheses about control of medically relevant phenotypes, such as treatment response or metastasis (21).

## USAGE

A flowchart summarizing the steps involved in using TMA Navigator (www.tmanavigator.org) is given in Supplementary Figure S1 and includes embedded hyperlinks to relevant parts of the user guide. Extensive help documentation is available by clicking on the *Help* button near the top-right of any page on the website, which opens at the section relevant to the current page. Many parts of the website have context-sensitive help, including tooltips and links from headings to appropriate subsections of the user guide. The first step in working with TMA Navigator is to create a dataset by importing marker scores, typically protein expression values; survival information can also be uploaded. A unique page for the dataset (the ‘dataset page’) has a *Run analysis* button providing access to data exploration, network inference and survival analysis. Analyses are processed in a queuing system and results are accessed from the dataset page.

### Importing data

TMA Navigator has a button labelled *Add dataset* near the top-right of every page to start the process of importing marker data. A grid format is required, with markers as columns and samples as rows. Marker replicates are specified by multiple columns with identical names. File formats accepted are Microsoft Excel (.xls, .xlsx), tab-separated (.tsv, .txt) or comma-separated values (.csv). For anonymous guest users, an imported dataset receives a unique URL, which is easily bookmarked and protected by a random key. Alternatively, users may register an account, which provides a single point of reference for multiple uploaded datasets.

Tissue microarray datasets are often split across multiple TMA blocks, which can lead to unwanted non-biological variation (batch effects). TMA Navigator provides an option for batch effect reduction using ComBat (17). We have adapted ComBat for use with TMAs, including improved error handling and automatic removal of replicates/markers that prove problematic due to missing data. Batch correction is offered during data import when batch information is included with marker scores—batches are indicated by a column named *Batch and covariates specified with a column name including the prefix *cov. Additional information on batch correction is provided at www.tmanavigator.org/help/score-requirements#batches.

Survival data are uploaded using the *Attach survival* button located on the dataset page. Patient identifiers in the TMA marker and survival data must match; anonymous patient identifiers such as a sequential numeric value must be used. The user guide (www.tmanavigator.org/help) gives further details on data import and formatting requirements.

### Data exploration

Marker distributions may be visualized using density plots (continuous data) or histograms (categorical data). Samples may be clustered by modelling marker expression as a mixture of Gaussian distributions. The number of clusters is determined automatically, and the procedure is fully unsupervised (methods). The mixture model is plotted with the centre of each cluster indicated, overlaid with a density plot and histogram; model parameters are displayed in a sidebar. Risk stratification according to marker values is commonly done manually or with quantiles (4,28,29). Mixture modelling with appropriate regularization (methods) has significant advantages, providing fully automated and statistically well-founded identification of groups according to expression values. Marker relationships may be explored with a heatmap (Supplementary Figure S2).

**Figure 1.**
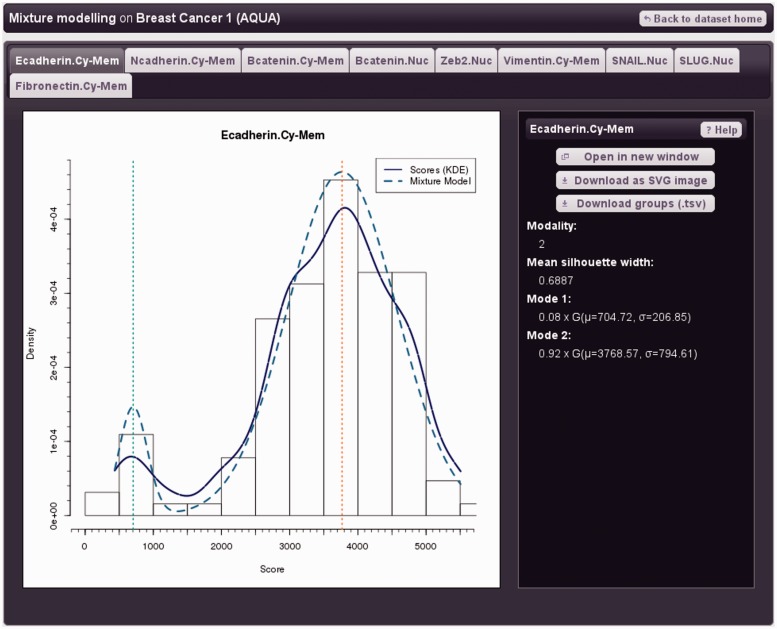
Gaussian mixture model of non-nuclear E-cadherin expression in primary invasive ductal breast tumours (demonstration dataset ‘Breast Cancer 1’). The histogram (black outlined bars) and kernel density estimation plot (solid blue line) both indicate protein expression. The mixture model is shown as a dotted turquoise line. Two patient groups were identified; the mean expression value for each group is shown by a vertical dotted line, and mixture model parameters are given on the right of the figure. The tabbed interface (top) allows easy navigation between markers.

**Figure 2.**
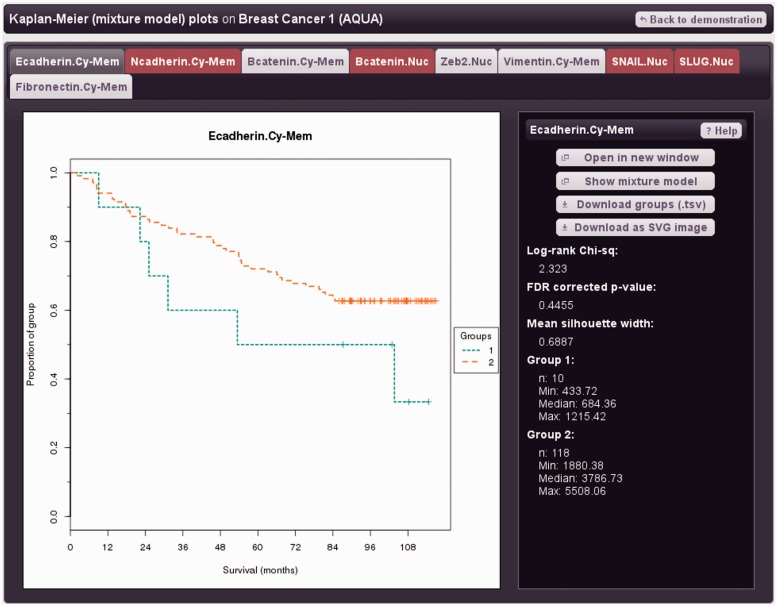
Survival analysis with E-cadherin expression informed by mixture modelling. Kaplan–Meier plot: x-axis denotes overall survival in months, y-axis the proportion of the group alive. Stratification of invasive ductal breast cancers by mixture modelling of E-cadherin expression (AQUA data); the low-expressing group shows a trend for worse prognosis consistent with expectations. Marker tabs shown in red indicate single group (unimodal) mixture models, for which Kaplan–Meier plots are not available.

Figure 1 shows a mixture model for the protein E-cadherin in the dataset ‘Breast Cancer 1’ (Demonstration data). The suffix ‘Cy-Mem’ indicates cytoplasmic and membrane expression values (i.e. non-nuclear). E-cadherin is a clinically important adhesion protein that is putatively down-regulated in epithelial to mesenchymal transition (EMT) and metastasis (30–32). Mixture modelling identified two groups, ‘E-cadherin low’ (*n* = 10, mean score = 705) and ‘E-cadherin high’ (*n* = 118, mean score = 3769). Survival of these groups was investigated in TMA Navigator (Figure 2); the ‘E-cadherin low’ group showed a trend for worse survival, consistent with expectations (28,31,33).

### Survival analysis

Survival analysis involves statistical testing to examine relationships of marker scores with survival, accounting for censoring, for a review see (34). Groups are defined according to marker scores with survival displayed as a Kaplan–Meier plot (19). The difference in survival between groups is tested for significance using the Mantel-Cox log-rank test (35) with false discovery rate (FDR) correction applied (36). Figure 2 and Supplementary Figure S3 show Kaplan–Meier plots for E-cadherin and PTEN expression respectively on ‘Breast Cancer 1’ (invasive ductal) and ‘Breast Cancer 3’ (trastuzumab-treated) cohorts (Demonstration data). Grouping according to E-cadherin expression (Figure 2) was determined by mixture modelling, a fully unsupervised approach (Data exploration). Loss of E-cadherin confers poor prognosis (30,31,33), and the low-expressing group showed the expected trend for worse survival. TMA Navigator provides for survival analyses on mixture modelling results as the option ‘Kaplan–Meier (mixture model) plots’ in the *Run analysis* dialogue box. Supplementary Figure S3 shows survival for tertiles of PTEN expression (FDR *P* = 0.0207), a tumour suppressor important for trastuzumab response (37) scored using the semi-continuous ‘quickscore’ method (Demonstration data). Splitting by tertiles provides roughly equal group sizes and so may improve prospects of obtaining statistical significance (38). However, these groups are unlikely to reflect modes of the underlying marker score distribution. Mixture modelling provides for biologically motivated grouping and so may enable better risk stratification, although associated smaller group sizes can lead to lower statistical power (38). When mixture modelling returns a single Gaussian (unimodal) model, survival analysis is still possible using tertiles. For categorical data, groups are defined by score values.

### Network inference

Correlation networks provide a useful abstraction of the relationships (edges) between multiple markers, for example to inform biomarker discovery (39). TMA Navigator is typically used for analysis of protein expression, although markers might also include clinical variables such as lymph node metastasis count. TMA studies usually involve relatively few proteins that may have close relationships in signalling and/or metabolic pathways; therefore, common assumptions about network structure such as sparsity (40,41) do not necessarily hold. Furthermore, TMA data are subject to multiple sources of confounding variation that may be extremely challenging to remove, including differences in surgical procedure, sample age, reagent batch/age, sample fixation and variation in the material analysed. This variation acts as ‘noise’ and may reduce correlation values even when markers have biological relationships (17). Accordingly, edge thresholding for TMA networks is usefully tailored to the individual dataset studied, and to enable this, TMA Navigator affords access to correlation values for all marker pairs. Statistical significance is normally applied to identify minimum threshold values (e.g. FDR *P*-value ≤ 0.05). Correlations can identify biologically meaningful edges (42,43); however, statistically significant correlations do not necessarily underlie genuine functional interactions (44). Ideally, the edge threshold may be calibrated against negative control markers unrelated to the pathway(s) studied, as well as positive controls where relationships are well characterized in the system of interest.

Correlation networks may be inferred in TMA Navigator using several measures: mutual information, Spearman correlation or Pearson correlation. Mutual information measures statistical dependency between markers and therefore detects many types of interaction, although does not distinguish between positive and negative relationships. Also, significance is estimated by permutation and therefore statistical power is influenced by sample size and dependencies within the data (45). Spearman and Pearson correlation are limited to detecting monotonic and linear marker relationships respectively, but have the advantage of analytical significance estimation (methods) and can identify signed edges. Interactive thresholding is available on *P*-values adjusted for multiple hypothesis testing [Benjamini–Yekutieli (46) or Bonferroni correction], displayed as an interactive network using the Cytoscape Web plugin (47).

**Figure 3.**
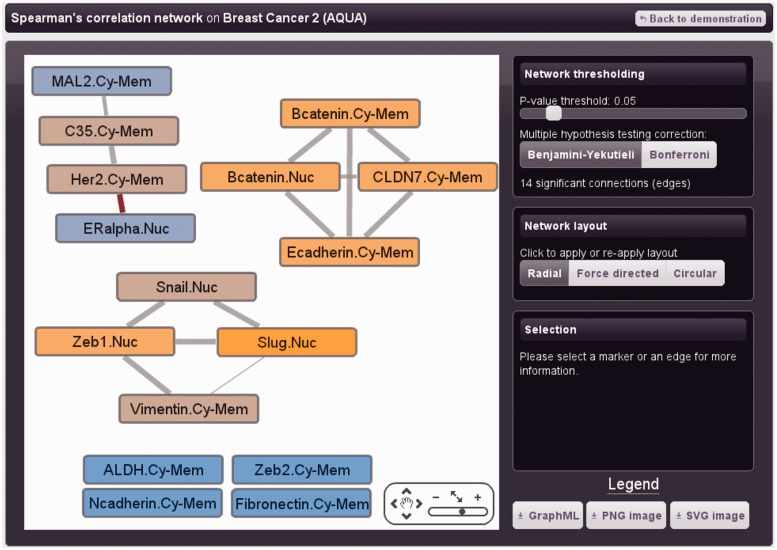
Spearman correlation network for trastuzumab-treated breast cancers. All marker pairs were scored using Spearman correlation and significant edges (FDR *P* ≤ 0.05) are shown. Colour of network nodes (markers) ranges from blue to orange, indicating low to high degree (number of significant connections). Positive and negative edges are respectively shown in grey and red. The network can be explored interactively, for example to alter layout and set significance threshold. Clicking on a marker summarizes neighbours, clicking on an edge displays the Spearman correlation and *P*-value. The above network recapitulates several expected interactions including a cluster of proteins that promote EMT (Snail, Slug, Vimentin, ZEB1) and an adhesion cluster (E-cadherin, β-catenin, Claudin-7) (30). Networks may also be exported as GraphML for use with external software or as a PNG or SVG image.

Figure 3 shows a Spearman correlation network for the dataset ‘Breast Cancer 2’ (Demonstration data), thresholded at FDR *P* ≤ 0.05 (46). Three components are identified, one (top-left) with the expected positive relationship between C35 and HER2 (48) and negative relationship between HER2 and ER-α (49). Interestingly, a positive relationship between C35 and MAL2 is found, in contrast to PCR results in cell culture with C35 induction (48). The second component (bottom) includes expected edges between the EMT transcription factors Snail, Slug, ZEB1 (30). The third component (top-right) includes edges between E-cadherin, Claudin-7 and β-catenin, as expected (30,48), suggesting a primary role for β-catenin in adhesion in this cohort, although an edge between nuclear β-catenin and Snail occurs close to the significance threshold (FDR *P* = 0.0783).

### Demonstration data

Several example datasets are available to demonstrate the capabilities of TMA Navigator (www.tmanavigator.org/demo). The dataset ‘Breast Cancer 1’ includes expression data for nine markers obtained using AQUA (16) and survival over 9 years for a cohort of 128 lymph node positive patients (10). The dataset ‘Breast Cancer 2’ has AQUA expression for 16 markers and survival over 5 years for a cohort of 92 trastuzumab-treated patients (37). The dataset ‘Breast Cancer 3’ includes expression for four markers measured using a semi-continuous approach and survival over 5 years on 122 trastuzumab-treated patients (37). The latter dataset has also been discretized into five quantiles for demonstration of categorical data handling. Antibodies for the above datasets are summarized in Supplementary Table S1; all data are from primary tumours. The example datasets described above are available pre-imported in TMA Navigator, and may also be downloaded.

## METHODS

Density plots approximate the empirical score distribution non-parametrically with adaptive bandwidth kernel density estimation (50,51). Mixture modelling identifies clusters of samples using expectation-maximization (52) to fit a mixture of Gaussian distributions to marker values. Each cluster has independent mean and standard deviation parameters, better aligning with biological expectations than fixed standard deviation. The number of clusters (modality) is selected using the Bayesian information criterion (BIC) (53). Survival is examined by Kaplan–Meier analysis (19), using the Mantel-Cox log-rank test (35), and stratification determined per marker with Benjamini–Hochberg corrected *P*-values (36). Network edge significance is determined using algorithm AS89 (54) (Spearman if *n* < 1290), Student t approximation (Spearman, Pearson) or permutation (mutual information), and *P*-values corrected with Benjamini–Yekutieli (recommended), or the overly conservative Bonferroni method (46,55). The service architecture is illustrated in Supplementary Figure S4 and described in Supplementary Data.

## CONCLUDING REMARKS

TMAs offer high-throughput immunohistochemical analysis of clinical samples and provide for study of tissue and cell-type specific networks underlying pathophenotypes (4,21). TMA Navigator is a unique interactive platform for TMA data processing and analysis that has been successfully tested on multiple web browsers (Internet Explorer, Firefox, Chrome, Opera, Safari). Key features include batch correction (17), unsupervised stratification by marker scores, survival analysis and network inference. An extensive user guide and demonstration datasets are available. We very much appreciate feedback on any issues relating to TMA Navigator, ideally sent via the form at www.tmanavigator.org/contact, and welcome requests for new functionality.

## SUPPLEMENTARY DATA

Supplementary Data are available at NAR Online: Supplementary Table 1 and Supplementary Figures 1–4.
